# The Active Inference Model of Coherence Therapy

**DOI:** 10.3389/fnhum.2022.955558

**Published:** 2023-01-04

**Authors:** D. Eric Chamberlin

**Affiliations:** Chamberlin Applied Neuroscience, Glastonbury, CT, United States

**Keywords:** Coherence Therapy, memory control and access, Bayesian Brain, Free Energy Principle and Active Inference (FEP-AI) framework, post-freudian psychology, psychotherapy, memory representation, conscious access

## Abstract

Coherence Therapy is an empirically derived experiential psychotherapy based on Psychological Constructivism. Symptoms are viewed as necessary output from an implicit model of the world. The therapist curates experiences and directs attention toward discovering the model. Rendered explicit, the model is juxtaposed with contradictory knowledge driving memory re-consolidation with resolution of the symptom. The Bayesian Brain views perception and action as inferential processes. Prior beliefs are combined in a generative model to explain the hidden causes of sensations through a process of Active Inference. Prior beliefs that are poor fits to the real world are suboptimal. Suboptimal priors with optimal inference produce Bayes Optimal Pathology with behavioral symptoms. The Active Inference Model of Coherence Therapy posits that Coherence Therapy is a dyadic act of therapist guided Active Inference that renders the (probable) hidden causes of a client’s behavior conscious. The therapist’s sustained attention on the goal of inference helps to overcome memory control bias against retrieval of the affectively charged suboptimal prior. Serial experiences cue memory retrieval and re-instantiation of the physiological/affective state that necessitates production of the symptom in a particular context. As this process continues there is a break in modularity with assimilation into broader networks of experience. Typically, the symptom produced by optimal inference with the suboptimal prior is experienced as unnecessary/inappropriate when taken out of the particular context. The implicit construct has been re-represented and rendered consciously accessible, by a more complex but more accurate model in which the symptom is necessary in some contexts but not others. There is an experience of agency and control in symptom creation, accompanied by the spontaneous production of context appropriate behavior. The capacity for inference has been restored. The Active Inference Model of Coherence Therapy provides a framework for Coherence Therapy as a computational process which can serve as the basis for new therapeutic interventions and experimental designs integrating biological, cognitive, behavioral, and environmental factors.

## Introduction to the Active Inference Model of Coherence Therapy (AIMCT)

Freud’s Project for a Scientific Psychology attempted to ground Psychology in the Natural Sciences (Freud, 1895). Steeped in physiology and Thermodynamics, Freud came to view living things as dynamic energy systems. He envisioned Psychology as explainable through the physiology of the brain (Cieri and Esposito, 2019). Ultimately Freud was forced to abandon the Project due to the technical and conceptual limitations of the time. This included what he considered “the heart of the riddle,” the physiology of the phenomenon of repression. In doing so he appears to have reluctantly adopted a Functionalist perspective that the mind can be studied independently of the brain (Johnson-Laird, 1983). Having abandoned pursuit of a deep connection to neurophysiology, Freud developed the meta-psychology that informs Psychoanalysis.

Recent developments have reanimated Freud’s vision of understanding Psychology as the physiology of the brain (Eliasmith, 2003). The identification of resting state networks has facilitated a preliminary mapping from the psychological concepts of Psychoanalysis to the activity of the Default network (Carhart-Harris and Friston, 2010). This is part of a larger body of work known as Neuro-psychoanalysis (Solms and Turnbull, 2011; Johnson and Flores Mosri, 2016). Extending beyond Psychoanalysis, network function has been used to model the pathophysiology of Post Traumatic Stress Disorder (PTSD), and to its resolution with Eye Movement Desensitization and Reprocessing (EMDR) (Lanius et al., 2015; Chamberlin, 2019a). Viewed from this perspective the distance between the psychological and the physiological appears to diminish in what has been called Dual Aspect Monism. Briefly, “the mind and brain are emergent aspects of the same neuronal dynamics” (Hobson et al., 2021).

Another recently developed tool is the Free Energy Principle, and its realization in the Bayesian Brain (Friston and Stephan, 2007; Friston, 2009; Holmes and Nolte, 2019). The Free Energy Principle as developed by Friston et al. is an information theoretic isomorph of Statistical Thermodynamics that begins from consideration of “first principles.” That is, what a living thing must do to continue living (Clark, 2013). In order to exist a living thing must resist the dissipative forces of entropy by minimizing surprising, catastrophic exchanges with the environment e.g., being a fish out of water. To accomplish this the agent must reduce its Free Energy, which is essentially the error of its predictions (Hohwy, 2013). In other words, to stay alive an agent must have a model of the world that is capable of generating predictions about sensory data, and must minimize the error of those predictions (Friston and Stephan, 2007). The Free Energy Principle through prediction error minimization offers the possibility of explaining disparate psychological functions like perception, attention, and action with a conceptually simple mechanism that is physiologically plausible and empirically verifiable (Friston, 2010). The non-negotiable requirements of thermodynamics regarding energy and information apply at all levels of consideration e.g., from molecular to psychological (Strelnikov, 2014). This creates a common language or currency between disciplines that facilitates a deeper connection than simple associations between mind and brain (Clark, 2016). Thus the Free Energy Principle has been used to provide putative links from Psychoanalysis to neurophysiology (Hopkins, 2012, 2016; Johnson and Flores Mosri, 2016). Post-Freudian therapeutic approaches have also benefited from the explanatory power of the Free Energy Principle. For example, the Predictive Processing Model of EMDR suggests how the epistemic affordance of eye movements can be used to drive free energy minimization and belief updating in PTSD (Chamberlin, 2019b).

The Active Inference Model of Coherence Therapy attempts to leverage the explanatory power of the Free Energy Principle by offering critical analysis of an empirically validated post-Freudian psychotherapy through the lens of Bayes Optimal Pathology (Schwartenbeck et al., 2015). Application of Bayes Optimal Pathology presents two key questions for computational neuropsychology (Parr et al., 2018). The first question is “what are the prior beliefs that would have to be held to make this behavior optimal?” Next, having identified the (putative) suboptimal priors, the question becomes “what are the biological substrates of these priors”? Or, what happens in the brain? These questions will be addressed sequentially in what can overall be considered an exercise in Psychodynamic Neuroscience. In essence, using the Free Energy Principle as a bridge between mind and brain (Cieri and Esposito, 2019).

## Introduction to Coherence Therapy

Coherence Therapy (CT) is empirically derived experiential psychotherapy developed by Bruce Ecker, Laurel Hulley and Robin Ticic (Ecker et al., 2012). Based on Psychological Constructivism, the emphasis of Coherence Therapy is on the individual’s subjective experience of reality resulting from models built to understand the world, and to support successful action in it. To wit: “Perhaps most central to the constructivist vision is the contention that we do not passively perceive the world as it actually is, but rather shape and form what we know and experience through active, constructive mental processes” (Toomey and Ecker, 2007). And further, “…the function of forming “knowings” is to optimize the person’s adaptation in the experiential world, not to accurately discern the true nature of things.” With these assumptions Coherence Therapy is firmly embedded in the Constructivist tradition.

Coherence Therapy distinguishes itself from other psychotherapies with several additional assumptions. The first is that a client’s presenting symptom is assumed to be a logical, “coherent” product of the “implicit knowing” or construction of reality held by the person in the present (Ecker et al., 2012). Specifically, there is an implicit “emotional truth” that makes it necessary to have or produce the symptom (Ecker and Hulley, 1996). From this perspective, despite being undesirable, the symptom is not a “dysfunction” of the brain. Per Ecker et.al. “… a client’s seemingly irrational, out-of-control presenting symptom is actually a sensible, cogent, orderly expression of the person’s existing constructions of self and world, not a “disorder” or pathology” (Toomey and Ecker, 2007). The problem lies with the implicit model. The central task of the therapist is to facilitate an experience of the implicit “emotional truth” becoming explicit.

Following symptom identification, Discovery is the phase of Coherence Therapy which attempts to curate experiences that render the emotion and implicit model of the world that necessitates symptom production, explicit (Ecker et al., 2012). Examples of experiences include symptom deprivation, sentence completion, role play, imaginal interaction techniques etc (Ecker, 2016). The Principle of Immediate Accessibility asserts that the implicit model can be known rapidly (from one to several sessions depending on the underlying complexity of the material) (Ecker and Hulley, 1996; Ecker, 2018a). This stands in contrast to conventional wisdom in psychotherapy about the difficulty of reliably accessing unconscious material swiftly, and is based on Ecker’s research developing the experiential techniques that facilitate this process. It appears that the assumed coherence or logical necessity of the symptom given the implicit model facilitates efficient elucidation of the model (Ecker et al., 2012).

An important element of the retrieval of the implicit model or schema in the Discovery process is the sustained application of attention to the task by both parties, driven by the therapist (Ecker and Hulley, 1996). From the perspective of the client, the symptom is problematic and the idea that the symptom “makes sense” at some level is deeply counter-intuitive. Thus, the success of the Discovery process is contingent on the therapist’s commitment to the assumption of coherence to overcome the intuitive bias of the client and sustain focus on Discovery (Ecker et al., 2012). An additional challenge is that the implicit model is typically formed early in life in a situation with emotional urgency, often leading to repression or exclusion from consciousness of the emotionally charged memory (Ecker, 2015).

Following Discovery, the implicit model has been rendered explicit and the Integration phase ensues. Integration is intended to facilitate incorporation of the now explicit model into everyday awareness in preparation for the final phase of Juxtaposition (Ecker et al., 2012). In Juxtaposition the client is ushered into an experience of simultaneous awareness of the symptom necessitating model, and personally held contradictory living knowledge. This juxtaposition results in effortless cessation of the symptom going forward, having presumably driven prediction error mediated memory reconsolidation (Ecker et al., 2012). Typically, after having been rendered explicit the symptom is understood as being necessary in some contexts, e.g., past personal experience, but not in the present context (Ecker and Hulley, 1996). The result is an experience of agency in symptom production, and spontaneous context appropriate behavior thereafter.

### Discovery as the essential activity of Coherence Therapy

Thus far Ecker et al. have focused on the role of the Juxtaposition phase and presumed memory re-consolidation as being the core active element of this effective therapy (Ecker, 2018a). In contrast, this review will focus on the Discovery phase and its role in the therapeutic process. The reason for this is that in practice, successful Discovery of the precise symptom necessitating schema results in immediate and enduring cessation of the symptom in more than half of clients sampled (Ecker, 2018b; Ecker and Bridges, 2020). In these cases, it appears that when the symptom necessitating schema is first apprehended clearly in consciousness, the client is immediately confronted with contradictory knowledge that often leaves them bemused e.g., “When I think about it, that’s ridiculous” (needing to produce the symptom in the current context) (Ecker et al., 2012). Thus it appears that the Discovery phase is the only necessary and (often) sufficient phase of the process. While Integration and Juxtaposition likely ensure consistent, comprehensive resolution, something crucial appears to be happening in Discovery.

### The Bayesian Brain

The Bayesian Brain views perception and action as inferential processes (Doya et al., 2006; Parr et al., 2018). Recognizing that the brain does not have direct access to the world it must infer the hidden causes of the sensations it experiences. For example, light does not enter the brain, the inside of the skull is dark. Upon striking the retina, the energy of light is transformed into the firing of neurons. From the resulting patterns of neuronal firing the brain must infer, or guess, the hidden cause in the world eliciting that pattern (Hohwy, 2013). The process of inference is presumed to be accomplished by physiologically plausible computations employing Bayesian logic. The prior probability (the “prior”) is the probability of a cause before consideration of the particular sensory data. The likelihood is the conditional probability of the sensory data given the cause. Precision reflects confidence in prior beliefs and in sensory data, varying how much weight should be given to each in computations. Taken together the “prior” and the “likelihood” constitute a “generative model” i.e.,. a model of how the sensation is generated by a hidden cause in the world. This constitutes the brain’s “best guess” (as a probability) regarding the current state of the world.

### The Active Inference of embodied, embedded agents

Recognizing that inference occurs in an embodied agent, who is embedded in a particular environment in the world, it becomes apparent that it is an active process. In order to more accurately infer the causes of the sensations it experiences, the agent must act on the world and run experiments to test hypotheses about the hidden causes (Friston K. et al., 2012). For example, “what happens if I look over there, or push on this?” (Parr and Friston, 2017). The sensory results of such experiments provide data that is then used to revise current hypotheses and suggest new experiments. The best experiments resolve the most uncertainty, that is, provide the most information (Friston et al., 2016; Clark, 2018). For example looking toward the source of a sound is likely to provide more information about its cause than looking away from it. As this process of hypothesis generation, testing, revision, etc., proceeds, inferences about the current state of the world become more accurate. Formally, model evidence increases. This suggests that humans perceiving and acting in the world behave essentially as scientists. That is, from the perspective of Active Inference they employ the core elements of the Scientific Method as they learn about the world. This assertion is supported by a rich body of empiric work in childhood cognitive development (Gopnik and Wellman, 2012).

### Bayes Optimal Pathology

Human behavior is not always adaptive. How is this understood from a Bayesian perspective? The results of the Complete Class Theorem suggest that for any behavior, there is a prior belief that makes that behavior “Bayes Optimal” (Wald, 1947). This includes non-adaptive or pathological behavior. In other words, the inferential process itself may be intact i.e., “Bayes Optimal.” However, starting with a suboptimal prior, the behavior resulting from intact inference may be “pathological” (Schwartenbeck et al., 2015). By analogy, despite sound logic, if one starts with questionable assumptions the conclusion may be inappropriate. The result is Bayes Optimal Pathology. This suggests that a pathological behavior “makes sense” if we know the suboptimal prior belief it is based upon. It has been suggested that the identification of the suboptimal priors underlying pathological behavior is a promising clinical approach (Parr et al., 2018). If so, the question becomes “what are the prior beliefs that would need to be held to make a behavior Bayes Optimal”?

### Consilience of Coherence Therapy and Bayes Optimal Pathology

Coherence Therapy assumes that behavior, including pathology is always “coherent,” that is “makes sense” and is logically consistent if one identifies the underlying “emotional truth” or schema on which it is based. In fact, the underlying schema necessitates the behavior, including symptom production. This is analogous to the notion of Bayes Optimal Pathology where a suboptimal prior belief (=“emotional truth”) with intact Bayesian inference, gives rise to pathological behavior (a symptom). There is not necessarily “dysfunction” in the physiology of the brain (Parr et al., 2018). As suggested previously the question becomes “what are the prior beliefs that would need to be held to make the behavior Bayes Optimal?” The analogous question in Coherence Therapy is, “In what way does your (undesirable) behavior/symptom make sense?” The process of Discovery is essentially to curate experiences that lead to identification of the suboptimal prior in this particular client. This can be thought of as an act of meta-Bayesian inference in which the therapist is making inferences about the subject’s inferential process (Daunizeau et al., 2010).

### Discovery effects a representational redescription

The Discovery Phase of Coherence Therapy begins with a symptom that the subject has identified as problematic or undesirable. The shared initial goal as framed by the therapist is to identify the hidden cause of the client’s behavior, characterized as an implicit “emotional truth” or schema that necessitates symptom production. “The therapeutic target of change is a given symptom’s underlying emotional learning which is a schema, or mental model, that was learned long ago in an emotionally intense experience and has been controlling behavior and or state of mind from outside of awareness” (Ecker and Bridges, 2020). Of note is that the schema is not conscious despite its significant, problematic influence. Ecker et al. characterize finding this schema as “retrieval” of the schema. The Active Inference Model of Coherence Therapy (AIMCT) diverges significantly from this perspective viewing identification of the “symptom necessitating schema” as being the product of coordinated Active Inference that while often retrieving a relevant memory, always acts to re-represent the target schema at a higher hierarchical level. This process takes time as the back and forth exchanges of therapist and client establish the features of the representation. This new representation is consciously accessible and is available for verbal expression and different types of cognitive processing. This includes action (policy) selection. In addition, it is embedded in context and manifests agency. In other words, the essence of the Discovery phase of Coherence Therapy is knowledge construction through Active Inference. In the context of the Bayesian Brain applied to psychotherapy this type of knowledge construction has been called Structure Learning. It refers to “learning the repertoire or narratives that constitute our prior beliefs–or hypotheses–about how our world works, and how these might be influenced therapeutically” (Holmes and Nolte, 2019). Empiric simulations suggest that “structure learning can be seen as an emergent property of active inference (and learning) under generative models with “spare capacity”; where spare or uncommitted capacity is used to expand the repertoire of representations” (Smith et al., 2020).

### Convergence of Coherence Therapy and Psychoanalysis

Focusing on the notion of “representation” Hopkins synthesized the deep convergence of Freudian Metapsychology and the Bayesian Brain (Hopkins, 2012). Freud viewed symptoms as occurring in the setting of failures of First Person Authority (agency) due to emotionally conflicting representations held by the patient (Freud et al., 1961). The challenge of acting while holding conflicting representations was managed by suppression of one representation according to Freud. As in the Bayesian Brain, there can only be one best (highest probability) representation at a time. For example, in the binocular rivalry face-house image, the brain chooses the most likely representation at that moment and sees a face or a house, but not both. One representation is utilized while the other is suppressed (for the moment) (Hohwy, 2013). From Freud’s observations, however, the suppressed representation may exert effects on behavior that manifested as symptoms, without awareness of the role of the suppressed representation (This is consistent with continued evidence of the suppressed representation at lower cortical levels in binocular rivalry (Zhang et al., 2011)]. Utilizing observations of behavior and free association, the psychoanalyst infers regarding the “hidden cause” (suppressed representation) of the symptom delivering a verbal interpretation to the client that attempts to make this suppressed representation explicit and consciously accessible so that the conflict, and therefore the symptom can be resolved.

Although not its stated intent, the Discovery phase of CT often elicits suppressed memories as it “retrieves” the symptom necessitating schema, that is usually grounded in an emotionally intense childhood experience (Ecker, 2018a). Once explicit, the conflict and symptom are resolved with the emergence of agency, or what Freud called First Person Authority.

### The challenge of never represented “inchoate forces”

In addition to representations that were suppressed and therefore unavailable to consciousness, Freud also identified “inchoate forces” that had never been represented and yet had causal (behavioral) efficacy (Levine, 2012). Never having been consciously represented these implicit influences pose a significant problem for Psychoanalysis. The challenge of representing these influences verbally so that they can be thought, and processed further, has been called “the work of Psychic Figurability” (Botella and Botella, 2004). Because of the amorphous nature of these unconscious influences a significant concern with this work lies in ensuring that the jointly rendered explicit representation is the product of the client’s, and not the analyst’s mind, as much as possible (Levine, 2012). A further technical challenge is the required level of therapist activity which deviates significantly from the core Psychoanalytic practice of observation.

The AIMCT argues that Coherence Therapy addresses the challenge not only of rendering suppressed representations explicit, but also making those that have never been represented, explicit. It achieves this through coordinated active inference that utilizes therapist observation to make inferences that are utilized not as interpretations, but to design experiments that engender visceral experience and prediction error constrained disambiguation of hypotheses regarding the hidden causes of the client’s behavior. In the process a representational redescription is effected. Because therapist inference is only used to design disambiguating experiments, therapist bias and “contamination” are (hopefully) minimized. “To do Coherence Therapy is to say and do nothing designed to directly counteract, overcome or prevent the symptom, and to do no interpreting” (Ecker, 2016). “Better narrative, clever reframes, positive beliefs and other “counteractive” interventions are precluded” (Ecker et al., 2012). This helps to operationalize the constructivist assumption of “the client as the expert on their life,” with the therapist coming from a “not knowing” but actively curious position (Anderson, 1992). Requiring therapist inferences to be utilized only through experimental design helps to create a “firewall” that attempts to keep therapist models separate from the client’s (evolving) model. It likely contributes to the striking claim that “any competent (Coherence) Therapist should discover the same set of constructs” (Ecker and Hulley, 1996).

### Divergence of Coherence Therapy from Psychoanalysis

Like Psychoanalysis, Coherence Therapy makes extensive use of observation. However, unlike Psychoanalysis it is an intrinsically active therapy that uses experiments to create experiences and disambiguate competing hypotheses (Ecker, 2016). From the AIMCT perspective it is an iterative process where the therapist inverts his evolving generative model of the client’s (hidden) generative model to specify the subject’s likely suboptimal priors which are then subject to experimentation. This strategy is consistent with the suggestion that identification of possible suboptimal priors for experimentation is a starting point for phenotyping in Computational Psychiatry (Schwartenbeck and Friston, 2016). The core divergence of these two therapies is captured in the rough analogy: Psychoanalysis is to Perceptual Inference as CT is to Active Inference. That is, passive observation is replaced by active exploration and experimentation. While Perceptual Inference operates on latent states that cannot be controlled, active inference operates on states that can be controlled e.g., actively guiding another’s experience to gain a (mutual) understanding of why someone does something (Frith, 2012). Understanding how such representations may become explicit requires a deeper look at the representational redescription Model.

### Representational redescription begins with implicit representations

The model of representational redescription was developed to explain observations of cognitive development in children as they acquire language and solve problems (Karmiloff-Smith, 1979). As children learn they appear to pass through several stereotyped phases beginning with implicit representations that reflect knowledge of a procedure. That is, they are able to produce a particular output in response to particular external stimuli. However, while their behavior suggests that they know “what to do” in a given situation, they are unable to describe what they are doing, or why. At this level of implicit representation, “it is knowledge in the system, but it is not yet knowledge to the system” (Clark and Karmiloff-Smith, 1993). As a result, the knowledge that is embedded in the procedure is not available to other processors or the system as a whole. In that sense the implicit knowledge is “modular” (Karmiloff-Smith, 1994).

### Implicit representations are serially re-described and rendered explicit

The representational redescription model posits a progressive “explicitization” of the initial implicit procedural representation (Karmiloff-Smith, 1986). Based on empiric observations, the essential point is that the implicit representation is not rendered explicit as a result of simple “reading” by a conscious or semantic processor. Explicitization is thought to be a constructive process that takes time and often requires some type of “scaffolding” or “cognitive processing prop” to be effected. Successive iterations generate increasingly more likely representations as hypotheses are tested and revised (Gopnik and Wellman, 2012). An analogy is drawn to scientific discovery with the accretion of knowledge and progressive re-description resulting in a flexible knowledge structure that can be utilized in different ways. Recent work on the optimization of knowledge construction sheds light on the products of this process suggesting that the most useful schemas are composed of a balance between generalized semantic and detailed episodic memories (van Kesteren and Meeter, 2020). The AIMCT posits the Discovery Phase of Coherence Therapy is a process of progressive representational redescription that yields a relatively optimized schema with a balance of semantic generalization and episodic detail that typically renders the presenting symptom unnecessary, or inappropriate in the current context.

### Learning to be conscious

The concept of representational redescription has been incorporated into recent work that sheds light on the dynamics of the process of redescription. The Self-Organizing Meta-representational Account of consciousness (SOMA) suggests that consciousness is something the brain learns to do (Cleeremans et al., 2020). Driven by the need to manage the consequences of its actions on the world in order to ensure survival, the brain develops a model of itself. Starting with neuronal activity which is intrinsically non-conscious, the brain learns how to execute an action in response to certain stimuli without awareness of what it is doing. For example, a young child may have a sensation of fear and complain of a (non-existent) stomach ache. This results in staying home from school and avoiding a feared experience. There is not necessarily any awareness of this “procedure” or why it is executed, it just works. The uncomfortable sensation of fear goes away. According to SOMA this type of procedure may operate automatically without awareness, indefinitely as long as it is adaptive. However, if the consequences of the behavior become sufficiently problematic e.g., sensing irritability from an inconvenienced parent, the procedure may no longer be adaptive. Then the brain is doing something in its exchanges with the world that has unexpected, undesirable consequences. In other words, the brain’s activity is resulting in excessive uncertainty, and in preferred states not being realized. The brain may attempt to reduce uncertainty, and gain increased control by creating a model to explain the causes of the unexpected consequences. It does this by creating a representation of its own activity. The “procedure” with its initial sensation of fear, the act of verbalizing “stomach ache,” and the effects become objects for representation. “I feel fear, say I have a stomach ache, miss school, the fear goes away, and mom is irritated.” The brain has made its activity an object for itself to be represented. It is creating “a theory about itself” and in so doing there is the emergence of phenomenal consciousness, agency, and control (Cleeremans et al., 2020).

### The emergence of a self with agency

In the process of representing the brain’s activity to itself, the notion of a “self” necessarily emerges. To have a sensation, requires that there is an experiencer of the sensation, that is, a “self” that is having an experience (Fleming, 2020). And if an action is taken, there needs to be an agent taking that action. Thus the act of representation results in conscious awareness of a self with agency. Because this self can take action or not, there is the possibility of control.

The process of re-representing lower hierarchical levels can continue as needed to reduce uncertainty and increase control. For example, the child who has represented the original procedure may say “I was scared and said I had a stomach ache, missed a lot of school and mom got irritated” as an early representation. This may evolve into a higher level representation that is both more detailed, and more general e.g., “I was scared because I had a math test on those days, and I had failed one early in the year. When I’m scared, I avoid things.” And then a higher level “I was scared and avoided math tests because I really want to be an engineer, like my mother. So in order to do that I have to try to stop avoiding.” Of note is that one can easily imagine how this iterative re-representation could be facilitated by attentive, interested others who help “connect the dots” of self-experience e.g., parents, teachers etc.

There are several important things to note about this process. Learning to be conscious through representational redescription takes time and involves processing different types of information (perceptual, episodic memory, general knowledge etc.). This implies that the modularity of the initial implicit knowledge as noted by Karmiloff–Smith has been broken, consistent with the Global Neuronal Workspace Hypothesis. Per Dehaene et al. “For the GNW, consciousness serves a function: it evolved to break the modularity of non-conscious processing and broadcast information to a brain-wide network that makes information globally available for report (motor or verbal) and post-perceptual cognitive processing like working memory or decision making (Panagiotaropoulos et al., 2020). Also of note is that there is a progression toward models of increasing temporal depth driven by the need to reduce uncertainty and to realize preferred states. With the initial procedure there is no representation of consequences or future states e.g., mom getting irritated over time. As the re-representation process continues with additional iterations and elaboration of hierarchical levels, the expected consequences of actions are represented over progressively greater intervals of time. These inferences about the future e.g., what happens if I do this, or if I do that, are the province of Expected Free Energy.

### Models of the future-expected free energy

In the Bayesian Brain the process of belief updating is driven by the imperative to minimize the discrepancy between the prior belief or model and the observed data in order to survive (Hohwy, 2013). Too great a discrepancy or divergence between the belief and observation may be incompatible with life e.g., a fish that “expects” to be in water observing that it is not. This divergence or surprise cannot be computed directly, however, variational free energy or prediction error can be computed and serves as a bound on surprise i.e., Free energy is always greater than surprise (surprise or surprizal is used here as a formal construct of information theory, not in its colloquial sense.). Therefore, in active Inference the drive is to minimize surprise by minimizing (average) Free Energy (Friston, 2009). In the moment this can be accomplished by updating the model to bring it in to accord with the observations, or by taking action and sampling the world to obtain different observations that are closer to those that are expected. Either approach may minimize the free energy or prediction error.

Given that an agent must minimize Free Energy over time, taking action in the world entails additional challenges. Taking action implies choice i.e., to do this or that. And each choice has consequences that will unfold in the future. Therefore, in order to minimize Free Energy an agent must be able to model different counterfactual scenarios and their associated “expected free energy” (Parr et al., 2022). Such imagined sequences of actions are “policies,” and choosing between policies based on their expected free energy is Bayesian Model Selection. In generating these scenarios of possible action sequences and their consequences, one is creating “Memories of the Future” (Ingvar, 1985). This function of imagining and planning actions is thought to depend on the on the prefrontal cortex (Schacter et al., 2012; Fuster and Bressler, 2015). As such there are implications for this capacity as a function of stress and of development (Arnsten et al., 2015).

### Intractable complexity as mental disorder

Using Ainsworth’s Strange Situation Hopkins illustrates the concept of computational complexity as Mental Disorder (Hopkins, 2016). Placed in a room with mom and toys, infants happily explore and play. Then mom leaves the room and the infant is left with a stranger experiencing unexpected uncertainty. Upon mom’s return, the unsettled, surprised infant needs to minimize uncertainty. Attachment to mom prompts a kinematic trajectory of approach. Anger toward mom for abandonment prompts a trajectory of avoidance. Securely attached infants approach mom, express anger, accept comforting, settle quickly, and resume play. Those with disorganized attachment are unable to manage the computational complexity, and enact an incoherent kinematic trajectory with elements of approach and avoidance. In other words, when faced with different possible courses of action, each with it’s own uncertainty, the computations necessary to select a single, best course of action “seize up.” The resulting behavior reflects the inability to choose, and they remain in an unsettled state for a prolonged time. Per Hopkins: “The complexity of affordance competition and action selection in this basic social decision is so magnified by conflict as to render the required computations too emotionally complex for their generative models to manage.” Furthermore “the conflicts and traumas that Freud thought responsible for recourse to phantasy/virtual reality in mental disorder should be seen as forms of neurocomputational complexity, and that mental disorder is the product of such complexity together with the mechanisms that have evolved to reduce it.”

### Self-knowledge as inference

Clinical presentation often takes the form of “something is happening to me and I want it to stop.” As observed by Freud there is typically a failure of First Person Authority, or absence of agency. It is happening “to me.” The therapist’s assumption in Coherence Therapy is that the client is producing the symptom because it is necessary according to his current construction of reality (Ecker, 2016). The accumulation of evidence to support this belief requires exploration and development of the client’s self-knowledge.

From the perspective of Active Inference, insight or explaining one’s actions can be regarded as the product of inference (Parr and Pezzulo, 2021). Attempting to explain one’s behavior retrospectively requires comparison of different policies (sequences of actions) and their expected consequences. Based on the observed consequences one can select the policy that best explains the data. “I did that because I believed that this would happen.” This is analogous to *post hoc* model selection in science where experimental outcomes are used to disambiguate competing hypotheses yielding inference to the best explanation. Agent based simulations using this framework have produced some interesting results.

Parr and Pezzulo’s findings support their hypothesis that insight is confabulation that is constrained by data to a greater or lesser degree, depending on the quality of the data used to formulate the explanation (Parr and Pezzulo, 2021). Furthermore they observed the emergence of replay, an analog of episodic memory which is both declarative, and embedded in a context. Taken together these findings suggest that the quality of insight resulting from inference varies widely and improves with episodic declarative memories that are embedded in context.

With these observations in mind the Coherence Therapist begins Discovery with the client having essentially no insight. “This symptom is happening to me for no clear reason.” Therefore the self-knowledge inferential process begins with a therapist assumption that attempts to increase the client’s hypothesis space to include the possibility that the symptom is logical and that there is a clear (hidden) cause that involves self. In other words, a new hypothesis needs to be added to the subject’s model in order to adequately account for current observations (Smith et al., 2020). The client is invited to enter and explore the space, and a Duet for One ensues (Friston and Frith, 2015). This leads toward synchronization of the therapist and client model of symptom causality, driven by the high precision (confidence) of the therapist’s belief that the symptom is a logical product of the client’s self, driven by a particular construction of reality. This is a crucial step in repair of the client’s capacity for inference on this issue.

The process of Discovery employs a well-defined methodology, but is not prescriptive and does not have a protocol *per se* (Ecker, 2016). Rather the guiding imperative is Active Inference to the best explanation. The principal question is “What model causes production of this symptom?” All actions taken by the therapist including asking questions, suggesting experiments, clarifying responses, reflecting back observations of behavior etc., are designed to create epistemic affordances that reduce uncertainty and answer this principal question. Bayesian model selection is repeatedly enacted as the therapist considers and chooses which policies (experiments) will provide the greatest information gain and maximize reduction in uncertainty (FitzGerald et al., 2014). This process is analogous to the Scientific Method as experiments are chosen for the highest likelihood of disambiguating between competing hypotheses. The absence of a protocol in the crucial Discovery phase of CT may seem surprising, however, is supported by recent Active Inference simulations of linguistic dialog. To wit: “… the purposeful, inquisitive and abductive behaviors are all emergent properties of minimizing (expected) free energy. In other words, there is no need to handcraft any rules…” (Friston et al., 2020).

Consider a commonly employed experiment in Discovery. “Imagine being in the situation where the symptom typically occurs, without the symptom” (Ecker, 2016). *A priori* this is a high yield experiment as it offers an epistemic affordance as well as potential pragmatic (therapeutic) value. Recent empiric work in Endogenously Generated Emotion (EGE) suggests that simulation of a novel experience activates the Salience Network (correlating with subjective core affect) and the Default Mode Network (correlating with the generation of representations) coordinated by the Frontoparietal Control Network. This results in an affectively charged experience of something that has never occurred (Engen et al., 2017). With memory of actual events being the substrate of simulation (Schacter et al., 2007; Buckner et al., 2008), there is a sleight of hand in this Discovery experiment in that the client is potentially guided to remembering and re-experiencing a repressed memory. The AIMCT posits that memory control bias against recall and reexperiencing of the associated physiological state, is a common cause of impaired inference that precludes representational redescription, experience of agency and contextual embedding. Policy selection in the moment of a childhood, emotionally urgent situation is unavailable for revision and optimization. As a knowledge construct, the schema lacks the desirable attributes of context or episodic detail and functions as a suboptimal prior giving rise to Bayes Optimal Pathology.

### What does the Active Inference of Coherence Therapy look like?

Lacking an explicit protocol, the Discovery Phase of CT is challenging to describe. A useful analogy for the process is that of a Forensic Sketch Artist. Beginning with a sparse description from a witness, the artist renders an image or “prediction” of what the suspect looks like. The witness gives feedback on the image e.g., “the nose is too large and the lips are too thin.” The artist uses this feedback or prediction error to revise the image. Successive iterations of this process minimize the prediction error until a satisfactory, detailed representation is obtained. In a similar fashion the Coherence Therapist collects verbal reports, observations of somatic behavior, associations, results from experiments etc., and sketches the emerging model that is then subject to error correction by the client. While clients typically have great difficulty in articulating what the model is initially, they have little difficulty in identifying what is wrong with a proposed model, thus driving effective revision by the client-therapist system. This is consistent with the constructivist notion that in order to define what something is, one must define what it is not (Kelly, 1991). And as the emerging model approaches the client’s implicitly held model, there is robust affective resonance. “That’s it exactly!… that’s how it works!… my body is vibrating”…”I can’t help but smile” etc. (Ecker et al., 2012). Such affective resonance with involuntary activation of facial musculature has been captured in the clinical concepts of “limbic music” and “the face of fluency” (Murray, 1992; Topolinski et al., 2009). These responses are consistent with the reward of reduced uncertainty and structure learning that leads to “a ha!” or “eureka” moments of insight (Friston K. J. et al., 2017; Friston et al., 2020).

### Discovery resembles simulated annealing

The Active Inference of Discovery as described above is essentially a search for the implicit model driving symptom production. The goal is to find the model with the greatest evidence.

Model evidence can be decomposed into accuracy minus complexity. Therefore maximizing model evidence requires attention not only to accuracy, but to complexity as well. There is a cost for complexity. Overly complex models are not useful. The Discovery process, roughly captured in the Forensic Sketch Artist analogy above, progressively optimizes model evidence with shifts in hypotheses that capture and lose accuracy, and increase and decrease complexity. Essentially this is the application of Occams’s razor, or the principle of parsimony. As attributed to Einstein, “everything should be made as simple as possible, but not simpler.” For example an overly parameterized model will be rejected by the client when it doesn’t fit a particular instance that comes to mind i.e., it doesn’t adequately generalize. As a result, parameters will be pruned and the model subject to re-testing. The more iterations, the more likely the global minima of free energy is found. This process is analogous to simulated annealing in computer science where broad “high temperature” searches gradually give way to more narrow “low temperature” searches as the optimal solution is approached (Gopnik et al., 2017). In this process the therapist plays an important role in regulating the “temperature” of the search, beginning by encouraging broad exploration in an apparently empty hypothesis space- “I don’t see how this symptom could make sense, but okay, I’ll try….”

### Toward a process theory-how might it work in the brain?

Application of Bayes Optimal Pathology presents two key questions for computational neuropsychology (Parr et al., 2018). The first question is “what are the prior beliefs that would have to be held to make this behavior optimal?” The AIMCT has argued that a core activity of CT lies in answering this question through active inference and representational redescription that leads to Structure Learning. Having identified the (putative) suboptimal priors, the next question is “what are the biological substrates of these priors”?

Active Inference provides a principled framework for understanding behavior. As with any normative theory, its ultimate utility depends on how well it explains empiric observations and generates testable predictions (Bowers and Davis, 2012). Specifically, this means generating hypotheses grounded in physiologically plausible mechanisms that are answerable to empiric data, so called process theories (Parr et al., 2022). Process theories attempt to offer an answer of how the phenomenon in question actually works in the brain with specific references to anatomy and physiology. Recent work offers preliminary support for some process theories suggested by Active Inference (Friston K. et al., 2017). What follows is a preliminary sketch of a process theory regarding the genesis of suboptimal priors, including the apparent compromise of the inference process that precludes optimization of these priors. How might people get stuck with beliefs that are resistant to change and cause symptoms? What structures and mechanisms might be involved?

### Memory suppression regulates affect

Freud postulated that painful, unwanted memories could be excluded from awareness *via* a process he called Repression (Freud et al., 1961). Utilizing fMRI Anderson et.al. confirmed the existence of an “active forgetting process” he called suppression, that established a framework for the study of “motivated forgetting” (Anderson et al., 2004). To remember entails some degree of reactivation of the physiology, and re-experiencing of the associated affect (Daselaar et al., 2008; Danker and Anderson, 2010). Memory is thus an important source of endogenously generated emotion (Engen et al., 2017). As such it has been suggested that memory control is an important mechanism of affective regulation (Benoit et al., 2014; Catarino et al., 2015; Streb et al., 2016; Engen and Anderson, 2018; Mary et al., 2020). However, while suppressing retrieval of a memory may help to regulate affect, there appear to be costs as well (Smith et al., 2016).

### Memory suppression interferes with representational redescription

Suppression of memory retrieval is thought to utilize the right dorsolateral prefrontal cortex to dynamically inhibit hippocampal activity while simultaneously inhibiting extra-hippocampal regions supporting the retrieval of affect (amygdala) and scene features (sensory cortex) (Benoit and Anderson, 2012; Benoit et al., 2014; Depue et al., 2015; Gagnepain et al., 2017). The parietal cortex is believed to be a key intermediary in episodic retrieval suppression, as it is in retrieval (Shimamura, 2011; Paz-Alonso et al., 2013). Suppression of the retrieval process thus disrupts the reconstruction of the episodic memory by inhibiting the hippocampus, amygdala and sensory cortex. There is evidence to suggest that retrieval suppression with reduction in hippocampal activity may result in a “virtual lesion” that leaves an “amnestic shadow” beyond the repressed memory extending to memories that are close in time to the suppression (Hulbert et al., 2016). It also appears to render the related information unavailable for further processing e.g., representational redescription and inference. This is consistent with Freud’s notion of a repressed memory that maintains causal efficacy driving symptom production outside of conscious awareness.

### Recruiting motivation to recall a repressed memory

Typically forged in childhood in an emotionally urgent situation the “emotional truth” or implicit schema is often associated with a painful memory that has been suppressed. Accessing the memory is avoided because it is painful (Ecker, 2015). However, the presenting symptom is also problematic, if not painful. When placed in the framework of the AIMCT, the discomfort of the symptom drives exploration leading toward the retrieval of the suppressed memory and associated schema. In this context the suppressed memory is not simply a painful (re-) experience to be avoided, but a potential epistemic affordance with pragmatic utility. Retrieval of the experience may facilitate representational redescription of the implicit schema with the emergence of agency and therefore control over the symptom. This shift in memory control reflects a crucial aspect of Active Inference and the Bayesian Brain: we are in charge of selecting the data we sample in trying to adapt to our world (Pezzulo et al., 2018). And the choice of what memories are sampled appears to play a significant role in the pathogenesis of suboptimal priors. Coherence Therapy appears to recruit motivation to retrieve the painful suppressed memory thus making a different choice that leads to repair of the inference process.

### Motivation drives the approach or avoidance of knowing

Recently the theory of epistemic motivation has been integrated with active inference dissolving the dichotomy between motivation and cognition (Kruglanski et al., 2020). As the quintessential mechanism of knowledge construction, inference is argued to be suffused with motivation (Pezzulo et al., 2018). The essence of this idea is that motivational bias is present in the choice of policies pursued for epistemic affordances. Sometimes we want to know e.g., “I think I did well so I’m going to try to find out what grade I received.” This leads to behavior that provides epistemic affordance e.g., going to the classroom where the grades are posted. At other times we don’t want to know e.g., “I think I failed so I’m going to get something to eat.” In this case a policy is chosen with no apparent epistemic affordance regarding the grade. More accurately, ignorance is pursued as the desired state. Of note is that the policy also reflects motivation to realize a “not hungry, satisfied state.” The epistemic affordance pursued is regarding food, not grades. From this perspective motivation plays a crucial role in epistemic foraging and therefore the knowledge construction resulting from active inference.

Utilizing the Structural Model of neocortical organization (Barbas and Rempel-Clower, 1997) the Adaptive Bayes Process Model suggests how relatively undifferentiated limbic cortex can influence highly differentiated hetero-modal cortex thus infusing motivation into the computations of the neocortex to facilitate Motivated Control (Tucker and Luu, 2021). Limbic and subcortical homeostatic control provide affectively charged expectancies which are incorporated to facilitate allostasis, the motive control of expectancies for future events. With this, the Adaptive Bayes Process Model suggests a neuropsychology to explain how motivation can be “baked in” to cognition and the Bayesian Brain.

Returning to Discovery in CT, the therapist’s high precision (confidence) belief that an implicit model necessitating production of the symptom can be found and will be useful provides motivation for active inference for both parties. To wit: “It emerged that the key condition for brief deep work was the therapist’s conviction that the unconscious constructs generating the client’s problem are immediately accessible and changeable from the start of therapy” (Ecker and Hulley, 1996). Recent empiric work suggests that such “…optimistic expectancies involve particularly strong predictions of reward causing automatic guidance of attention to reward…” (Singh et al., 2020). This is consistent with the idea that motivation influences epistemic foraging from the start (Kruglanski et al., 2020). In addition to the therapist’s optimism, the client’s subjective distress regarding the symptom provides motivation. Potentially these jointly held motivations can prevail over the drive to suppress a relevant memory. If so, how this might work in the brain.

### Attention drives low confidence memory search

The question “in what way does the symptom make sense for you?” invites a goal directed memory search. The Attention to Memory Model posits that the Dorsal Parietal Cortex is associated with the allocation of attentional resources to memory retrieval according to the goals of the person (top-down attention) (Cabeza et al., 2008). The dorsal parietal cortex is thought to modulate memory retrieval activity in the medial temporal lobe while the ventral parietal cortex detects when relevant memories have been retrieved, prompting a shift in attention to the retrieved contents (bottom-up attention (Corbetta et al., 2008). The dorsal parietal cortex is particularly important in effortful, “attention demanding low confidence searches” where the rememberer is unsure if they know something (“vaguely familiar”) (Ciaramelli et al., 2020). If memory retrieval is successful the ventral parietal cortex will capture attention to the MTL output with the experience of “recollection” with episodic details.

The challenge of a successful attention demanding low confidence search is reflected by the assertion that directing attention toward the coherence of the symptom (and its implicit model) is the central activity in Coherence Therapy (Ecker and Toomey, 2008). Therapist attention is important in part because the products of the search including associations, somatic expression etc., are often not recognized by the client as being relevant (Ecker and Hulley, 1996). Recall the progressive “explicitization” of Karmiloff Smith requiring “scaffolding” as the knowledge is re-represented into a new structure. In this “bootstrapping” process the client doesn’t know the structure he is building, and therefore can’t always recognize the products of the search as being “building blocks” of the new structure. Therapist attention to capture and reflect back the “building blocks” is thus crucial.

As noted previously attentional resources for recall are allocated according to the goals of the person. Autobiographical memories are multifaceted constructs that contain conceptual as well as perceptual details. Recall of autobiographical events are thought to occur on a gradient of abstraction from conceptual to perceptually based episodic memory as a function of the goal of remembering (Sheldon et al., 2019). This results from specialization with the anterior hippocampus being activated in conceptual recall, while the posterior hippocampus is activated in perceptual recall. And it has been suggested that being able to recall along a gradient of abstraction depending on the goal is important in maximizing the adaptive function of memory. For example, a memory that is recalled conceptually without grounding in episodic detail has the potential to be deployed in a context inappropriate manner (van Kesteren and Meeter, 2020). This is often the case with the suboptimal priors identified in Coherence Therapy (Ecker, 2016). In contrast, the AIMCT argues that the product of Active Inference in Discovery is a (relatively) optimized schema that is capable of generalization but is grounded in context/episodic detail.

Successful Discovery in CT yields an explicit schema that drives symptom production and is typically characterized by three elements: an emotional wound, a presupposition, and protective actions (Ecker and Hulley, 1996). Emotional wounds reflect a continuum of stressful experiences up to and including overt psychological trauma. Presuppositions are unexamined assumptions taken from experience that are part of the subjective model of how the world is, or how it works. Protective actions serve to avoid any unwanted experience or event and can take an extremely wide range of forms e.g., dissociation, compulsion, low self-worth, depression, shame etc. Note that protective actions include mental actions as well as overt physical action. From the perspective of the AIMCT the explicit schema is the suboptimal prior that both illuminates the compromise of active inference in the client, and provides a path to repair. To understand why, review of Active Inference under stress is helpful.

### Active inference and stress

Faced with a threat, an individual must respond to ensure survival. This requires calculating expected free energy including epistemic and pragmatic terms (Linson et al., 2018). The individual must consider different policies or sequences of actions that will minimize uncertainty through an optimized combination of exploration and exploitation leading to the preferred outcome. Then the best one must be selected. He must try to choose the most viable path to his desired future.

Faced with a potential existential threat e.g., an active shooter, short term thermodynamic considerations are paramount (Linson et al., 2020). The imperative is to reduce expected free energy quickly [Hamilton’s principle of least action (Parr et al., 2022)]. A policy to reduce uncertainty by exploration to better assess the threat runs the risk not only of putting oneself more directly in harm’s way, but also in wasting valuable time and energy that could be used for escape. Individuals differ in how much evidence they seek before abandoning assessment and trying to escape. More formally, the difference in the precision of prior preferences for avoiding harm is a sensory evidence accumulation threshold that serves as a crossover point from exploration to exploitation. At the crossover point the individual stops considering “what is it” and acts on the prior belief of mortal danger to “get safe.” Agent based modeling of this type of scenario has yielded some interesting results.

Agent based modeling of Active Inference under stress has been used to explore a model of PTSD (Linson et al., 2020). One important finding consistent with clinical phenomenology is that “a high negative preference (set by evolution or learning) for a stressor dependent outcome rapidly leads to exploitation (pragmatic foraging) before further state estimation.” The resulting exploitation has been called a “stressor mitigation policy.” “I really don’t want to experience this bad thing so I don’t bother to find out if I’m actually dealing with this bad thing, I just do this action to avoid the bad thing.” And crucially, this may lead to a fearful cue being taken not as a possible state of affairs prompting attempts to confirm, but as confirmatory evidence itself. The curiosity reflected by foraging behavior has been replaced by a state of pragmatic defensive responding.

The greater the balance is tipped away from exploration, the greater the compromise of the inference process. In this way PTSD has been conceptualized as being a condition of stress induced impairment on a continuum, where prior beliefs of threat are not subject to updating by sensory samples (Linson and Friston, 2019). Inference Interrupted.

The AIMCT posits the following scenario as the pathogenesis of the typical Symptom Requiring Emotional Truth retrieved in Coherence Therapy. In an “emotionally urgent situation in childhood,” the client experiences extreme surprizal (unexpected uncertainty) with formidable computational complexity that precludes optimal inference. High Nor-epinephrine compromises frontal lobe function including evaluation of context and planning (Schacter et al., 2012; Arnsten et al., 2015; Fuster and Bressler, 2015). Unable to infer to the contrary, the threat may be treated as an existential threat. The complexity of calculating expected free energy for different courses of action makes policy selection challenging, and a policy is selected (“a protective action”) to reduce uncertainty as rapidly as possible. (Free Energy reduction according to Hamilton’s principle of least action). The policy selected is an *a priori* high probability “sure thing.” In some cases, this may be an evolutionarily conserved response e.g., behavioral arrest or dissociation. A premium is placed on “never again” having that experience. If the “protective action” is successful, it persists as implicit procedural knowledge (Karmiloff–Smith Level I) to be used when needed. Computational complexity has been managed by a policy that requires minimal exploration and has minimal parameters e.g., no consideration of context. As a policy it is “good enough” (Gopnik et al., 2017) and is afforded high precision going forward because “it works” to avoid the dreaded situation.

The experience of extreme surprizal including physiological state, intractable complexity and negative affect is the “emotional wound” of the schema. Taken together these aversive elements prompt avoidance of the experience. This includes avoidance in thought. The individual avoids recall to be spared the interoception and pain of re-experiencing. Memory control blocks recall and the experience is unavailable for representational redescription. It remains implicit procedural knowledge in the system manifesting as automatic behavior when triggered by particular circumstance. Further Inference is precluded as the schema remains “modular” and isolated from other processors and types of knowledge. At that more benign end of the spectrum the schema may function as a suboptimal prior simply because it is never afforded the attention necessary for re-representation. “I never really thought it through.”

As suggested by the SOMA framework strong schemas may operate automatically outside of conscious awareness and control as long as the effects on behavior are adaptive (Cleeremans et al., 2020). However, with attention the contents of a schema may be brought to awareness to gain control when behavior is not adaptive. The AIMCT posits that the implicit schema adopted in an emotionally urgent situation in childhood operates automatically and is adaptive, until it isn’t. As the individual develops and matures her world becomes more complicated and circumstances change. From an objective function landscape perspective, the landscape has danced. What was once a local peak, a good enough solution, is now a valley. This necessitates search, and an increase in the complexity in the modeling of her world in order to remain a good regulator of that world (Conant and Ross Ashby, 1970). When that doesn’t occur due to blocked inference, unwanted consequences of her actions on the world begin to accumulate in the form of symptoms. Motivation to gain control builds. Coherence Therapy through an act of coordinated active inference repairs the inference process facilitating representational redescription with the emergence of context, agency and control.

### Clinical case vignette

JS was a 62 y/o woman who presented with decades of horrific compulsive nightmares while dreaming, and while awake. These involved being physically tortured, critically injured in a variety of accidents, or sexually assaulted. This occurred several times a week while awake and while sleeping. For example, contemplating a drive to visit a friend she would imagine her car breaking down and being sexually assaulted, killed, and stuffed into the trunk by a man who stopped to help. Multiple courses of psychotherapy, medication, and behavioral interventions over the years had not significantly impacted these symptoms, despite ameliorating debilitating anxiety and depression. Coherence Therapy was initiated with the goal of “understanding how these symptoms might make sense.” Discovery (active inference effecting representational re-description) yielded the following Emotional Truth (suboptimal prior). “My mother doesn’t protect me, so it’s critical that I imagine the worst case scenario so I am prepared for anything and can protect myself.” The emotional wound was a suppressed memory of terror as she was driven in a car by her intoxicated father, with her mother’s knowledge. The pre-supposition was “I have to do this by myself, no one else is going to help me.” And “I need to consider anything that might ever happen.” The protective action was to anticipate any and all possibilities that she could think of so she would have a plan for survival when she needed it, and would not be “caught off guard” (extreme surprizal) again.

Almost immediately following conscious expression the patient began to take exception with the presuppositions that contradicted her living knowledge. “My mother is deceased. I’m an adult and can protect myself. When my alcoholic ex-husband put my kids and I at risk I divorced him. I’ve been married for years to a loving husband who has helped me when I needed it. My life is good. I don’t need to do this anymore.”

Without further intervention the symptom frequency rapidly decreased over a period of weeks. The nightmares stopped. When the habitual thoughts did occur, they were rapidly followed by the thought “I don’t need to do this anymore” and ceased. She remains symptom free at 4 year follow up.

From the existing conceptualization of CT the implicit emotional truth was “retrieved” from “memory systems other than those that hold one’s explicit, autobiographical, episodic knowledge of past events” and made explicit (Ecker et al., 2012) (No physiological mechanisms or hypotheses offered to explain “retrieval,” how schema becomes explicit etc.) The schema was presumed to have been “locked away by extraordinarily durable synapses.” When the schema was confronted by contradictory living knowledge the mismatch triggered memory reconsolidation. This resulted in “unlearning” and “erasure” of the schema. “The process of unlearning and erasing that schema or mental model thoroughly resolves and puts to rest a core, personal theme of emotional distress….”(Ecker and Bridges, 2020). In summary, “the optimal process of psychotherapy consists of guiding the profound unlearning of the symptom- generating emotional learnings, nullifying and erasing them *via* the memory reconsolidation process” (Ecker and Bridges, 2020).

From the perspective of the AIMCT the client experiences a state of extreme surprizal with unexpected uncertainty (in a car being driven by drunk father). The computational complexity of calculating expected free energy to facilitate actions to ensure survival is formidable. Therefore a “stressor mitigation policy” is adopted to avoid ever finding herself in that situation again. This policy entails running simulations of an infinite set of “worst case scenarios.” It is deployed without epistemic foraging to determine if it is necessary (Am I actually in danger at this time, in this context?). As a child with an alcoholic father and a mother with a demonstrated failure to protect her, it is a “good enough” policy that is adaptive.

The experience was terrifying and the policy adopted serves to avoid having the experience again. This includes re-experiencing the affect of terror and the physiological arousal accompanying recall of this episodic memory. Memory control mechanisms presumably involving the right dorsolateral PFC suppress recall and help to regulate affect in an (initially) adaptive action. The policy continues to run outside of awareness. The unintended consequences begin to accumulate including nightmares and compulsive simulation of personal danger. The active suppression of recall renders the episodic memory unavailable for active inference and representational redescription.

The accumulation of unintended consequences (symptoms) motivates seeking treatment. Guided by the framework of Bayes Optimal Pathology the therapist assigns high precision (confidence) to the existence of a suboptimal prior and initiates active inference to identify the hidden causes of the client’s behavior. This is a goal directed “low confidence search” likely involving the frontal and dorsal parietal cortex. Motivation to eradicate the symptom prevails over motivation to suppress recall and the episodic memory is retrieved. “My father was drunk, it was terrifying.” Through selective activation of sub-regions in the hippocampus driven by the specific purpose of remembering, the appropriate elements of memory (perceptual vs. conceptual) are retrieved as the client’s brain re-represents its own activity in an attempt to understand and gain control over the unintended consequences of its actions. The result is a model of herself with agency and control over the production of her symptoms.

From the initial event to the present the client’s circumstances have changed significantly. The complexity of her situation has increased as she is now an adult with life experience, resources etc., that she didn’t have as a child. Therefore her (old) model is no longer a good regulator of her environment. Rapidly recognizing the shift in context (no longer a dependent child) the problematic behavior is recognized as unnecessary (I don’t need to do this anymore”). Rather than “unlearning” or “erasing” anything, she has learned a model of herself including motivation, behavior and consequences that contains an appreciation of its former utility and current irrelevance. This structure learning affords conscious access, agency, and control. And most importantly to the client, effortless cessation of the presenting symptom.

## Conclusion

The AIMCT places Coherence Therapy in the framework of the Free Energy Principle and the Bayesian Brain through the lens of Bayes Optimal Pathology. Coordinated Active Inference identifies the putative Suboptimal Prior in the subject leading to the repair of inference driven by reduction in free energy through representational redescription that confers conscious access, agency, control, and symptom resolution. In the process Coherence Therapy recruits the motivation to access painful memories. A process theory was proposed to explain how shifts in memory control leading to de-repression might occur given empiric data regarding motivated control, search and memory in the brain. Potential questions for future research that follow include: is there evidence of a R dorsal frontal activity shift as motivation is recruited and a suppressed memory is retrieved?, Is the dorsal parietal cortex engaged in the putative “low confidence search” for the suboptimal prior?

The identification of the Suboptimal Prior in a subject may also be used as a step toward identification of phenotypes. Suboptimal priors between subjects often show common features e.g., “occurring early in life, in an emotionally charged situation” (Ecker, 2016). Such observations can in turn be used to model phenotypes that are then subjected to *in silico* experimentation (Schwartenbeck and Friston, 2016). The results of such experimentation may then inform improvements in clinical practice e.g., extreme emotional stress may result in excessive precision of the suboptimal prior such that inference cannot be repaired through Coherence Therapy without modulation of the precision through neurotransmitter manipulation like dopamine blockade (Friston K. J. et al., 2012). In other words, some people may fail to benefit from this form of psychotherapy until a specific neurodynamic state is shifted pharmacologically. Thus, within the framework of Active Inference, the clinical practice of Coherence Therapy can help guide the research agenda for Computational Psychiatry, the results of which can inform better practice (Corlett and Fletcher, 2014). A virtuous cycle of Dual Aspect Monism where the emergent properties of mind and brain resulting from the underlying neural dynamics reflect and inform each other. Psychodynamic Neuroscience.

## Data availability statement

The original contributions presented in this study are included in this article/supplementary material, further inquiries can be directed to the corresponding author.

## Ethics statement

Written informed consent was obtained from the individual(s) for the publication of any potentially identifiable images or data included in this article.

## Author contributions

The author confirms being the sole contributor of this work and has approved it for publication.
